# Association Between Maternal Protein Intake and the Risk of Adverse Pregnancy Outcomes

**DOI:** 10.3390/nu18152543

**Published:** 2026-08-04

**Authors:** Suzette Rosas-Rogers, Ronald Anguzu, Alyssa M. Hernandez, Anna Palatnik

**Affiliations:** 1 Department of Obstetrics and Gynecology, Medical College of Wisconsin, Milwaukee, WI 53226, USA; alyhernandez@mcw.edu (A.M.H.); apalatnik@mcw.edu (A.P.); 2 Institute for Health and Humanity, Medical College of Wisconsin, Milwaukee, WI 53226, USA; ranguzu@mcw.edu; 3 Cardiovascular Center, Medical College of Wisconsin, Milwaukee, WI 53226, USA

**Keywords:** pregnancy, maternal diet, dietary protein intake, small for gestational age

## Abstract

**Background/Objectives**: This study examined the association between low protein intake and pregnancy outcomes to test whether low protein intake was associated with higher rates of small for gestational age (SGA), gestational diabetes mellitus (GDM), and hypertensive disorders of pregnancy (HDP), and lower rates of large for gestational age (LGA). **Methods**: A secondary analysis was performed using the Nulliparous Pregnancy Outcomes Study: Monitoring Mothers-to-Be (NuMoM2b). Participants missing diet data or pregnancy outcomes and whose pregnancy ended <20 weeks were excluded. Protein intake was calculated in grams per kilogram of bodyweight per day and stratified into quartiles. Multi-variable analyses were done to assess the association between protein intake quartiles and rates of HDP, GDM, SGA, and LGA, adjusting for relevant covariates. **Results**: Participants (n = 7067) were categorized into quartiles by daily protein intake: Q1 (<0.6 g/kg), Q2 (0.6–<0.8 g/kg), Q3 (0.8–<1.1 g/kg), and Q4 (≥1.1 g/kg). Lower protein intake was common among Black women (*p* = 0.001), women with less than a high school education (*p* < 0.001), unemployment (*p* = 0.004), lower income (*p* = 0.003), or government insurance (*p* = 0.04). After adjusting for age, insurance, education, income, chronic hypertension, and dietary kcal, protein intake resulted in significantly lower odds of developing SGA < 10th percentile for women in Q3 (aOR 0.78, 95% CI 0.61–0.99 for Q3). Protein in-take was not significantly associated with HDP, GDM or LGA. **Conclusions**: Moderate to high protein intake in the first trimester was associated with reduced SGA risk; however, these findings are exploratory and require confirmation in future studies.

## 1. Introduction

Nutritional requirements vary across the life course, with pregnancy representing a period of increased demand for both macronutrients and micronutrients to support maternal physiological adaptations and fetal growth [1,2,3,4,5,6]. Inadequate or excessive maternal nutrient intake is associated with adverse maternal and neonatal outcomes, including gestational diabetes (GDM), hypertensive disorders of pregnancy (HDP), anemia, fetal growth abnormalities and preterm birth [7,8,9,10]. While recommendations for adequate amounts of micro- and macronutrients exist, many pregnant women struggle to maintain a healthy diet that meets minimum daily intakes of micro- and macronutrients [7,11,12,13]. Macronutrients are essential to overall health and participate in nearly all biological pathways [14]. Protein, a key macronutrient, supplies amino acids that serve as fundamental building blocks for cellular structure and function and are critical for tissue growth and repair, protein synthesis, immune function, glucose and lipid metabolism, and energy production [14,15]. Adequate protein intake during pregnancy has been associated with reduced risk of fetal growth restriction, preterm birth, and low birthweight [16,17,18]. However, animal and human research studies have identified long-term health implications for offspring exposed to inadequate maternal protein intakes during pregnancy. Excessive and restricted maternal protein intakes during pregnancy have been reported to affect the developmental programming, epigenetic programing, growth, weight, and cardiometabolic health of offspring exposed to these conditions [16,19,20,21,22,23,24,25,26]. Nutrient intake recommendations are established using the estimated average requirements (EAR), which is the daily nutrient intake that meets the needs of 50% of healthy people in a specific population. An additional dietary reference is the recommended dietary allowance (RDA), which sets higher standards to encompass up to 98% of individuals by using the EAR plus two standard deviations [6,27]. The EAR for protein intake during pregnancy is 0.88 g per kilogram (g/kg) of bodyweight per day while the RDA suggests 1.1 g/kg of bodyweight per day [28,29,30]. However, the nutritional demand for protein intake increases in the second and third trimesters [6]. Few studies have examined macronutrient intake during pregnancy and their association with pregnancy outcomes, especially in the United States. The goal of this analysis was to examine the association between low protein intake and pregnancy outcomes, hypothesizing that low protein intake would be associated with higher rates of pregnancy complications such as SGA, GDM, and HDP, and lower rates of LGA.

## 2. Materials and Methods

This secondary analysis was performed using data from the Nulliparous Pregnancy Outcomes Study: Monitoring Mothers-to-Be (nuMoM2b), a large, prospective nulliparous cohort [31]. The original nuMoM2b study was a multi-site study that examined individual and environmental factors to identify characteristics that could predict pregnancy complications. The original dataset consisted of 10,038 nulliparous participants. This secondary analysis included 7067 participants who completed the modified Block 2005 Food Frequency Questionnaire (FFQ) [32]. Women with missing data on key pregnancy outcomes or with pregnancy loss prior to 20 weeks were excluded. The nuMoM2b dataset was accessed from the Eunice Kennedy Shriver National Institute of Child Health and Human Development Data and Specimen Hub (DASH), and the study was registered with our Institutional Review Board (PRO00051869, 30 May 2024).

The FFQ was administered at study visit 1, which occurred between 6 weeks 0 days and 13 weeks 6 days of gestation. The average daily intake of protein per participant was determined using the nutrient estimates diet analysis variable, labeled in the data set as “DTPROT”. The units for the reported diet analysis output variable were reported in grams. For this analysis, protein intake was calculated and stratified into empirical quartiles based on the grams per kilogram of bodyweight (first trimester visit) consumed daily. Quartile boundaries were based on RDA. Quartile 1 included participants that consumed <0.6 g g/kg of bodyweight per day, quartile 2 included 0.6–<0.8 g/kg, quartile 3 included 0.8–<1.1 g/kg, and quartile 4 was protein consumption ≥1.1 g/kg. The rational for these parameters was based on the RDA suggestion of 1.1 g/kg of bodyweight per day to meet the nutrient requirements of nearly all (97–98%) healthy pregnant women [6].

The following pregnancy outcomes were analyzed: (1) HDP, defined as gestational hypertension, mild and severe preeclampsia, eclampsia, HELLP syndrome and superimposed preeclampsia (preeclampsia diagnosed in women with chronic hypertension) [31,33,34]. (2) GDM, defined as occurring in the absence of preexisting diabetes and having abnormal values during glucose screen or glucose tolerance test; a GDM diagnosis is abstracted from the medical chart or White’s classification is used to define GDM by one of three glucose tolerance tests (GTT): fasting 3 h 100 g GTT with two abnormal glucose values ≥ 140 mg/dL for 3 h, ≥155 mg/dL for 2 h, ≥180 for 1 h, ≥95 mg/DL for fasting; fasting 2 h 75 g GTT with one abnormal glucose value ≥ 153 mg/dL for 2 h, ≥180 mg/dL for 1 h, ≥92 mg/dL for fasting; or non-fasting 50 g GTT ≥ 200 mg/dL if a 3 h or 2 h GTT was not performed [31,34]. (3) Large for gestational age (LGA), defined as birthweight > 90th percentile. And (4) small for gestational age (SGA), defined as birthweight ≤ 10th percentile [31]. To minimize the missing data for LGA and SGA, birthweight percentiles for gestational age and fetal sex were calculated using a United States (U.S.) birthweight reference for fetal growth [35]. The rates of all outcomes were compared between the quartiles of protein intake.

The following covariates were included in the analysis: maternal age (13–17, 18–34, 35–39, >40 years), maternal body mass index (BMI) category at visit 1 [underweight (<18.5 kg/m^2^), normal weight (18.5–<25 kg/m^2^), overweight (25–<30 kg/m^2^), obese (Class I) (30–<35 kg/m^2^), obese (Class II+) (≥35 kg/m^2^)], education level (bachelor’s or higher, some college/associate/technical degree, less than high school/no diploma), ethnicity (Hispanic/Latino or non-Hispanic), race (White, American Indian/Alaska Native, Asian, Native Hawaiian/Other Pacific Islander, Black/African American, more than one race, or unknown/not reported), income measured as a percentage of 2013 federal poverty level (<100%, 100–200%, or >200%), insurance (government, commercial, or self-pay), employment status, history of chronic hypertension, and the average daily total intake of kilocalories (kcal) labeled in the data set as “DT_kcal”.

**Figure 1:**
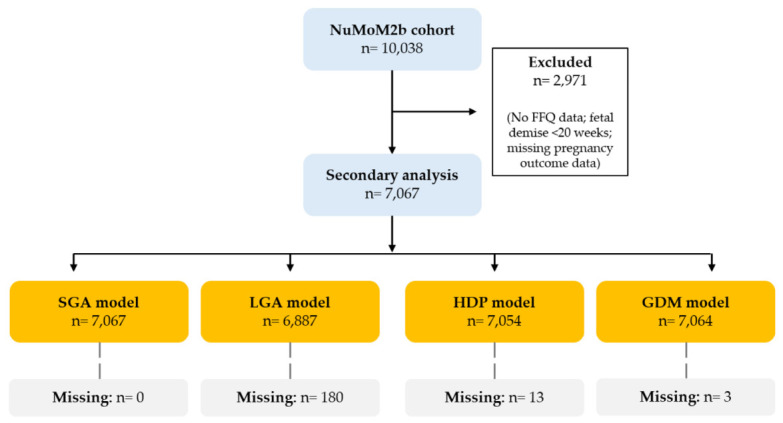
Participant flow diagram from the original NuMoM2b cohort through the analytic sample for this secondary analysis to each outcome-specific model. Of the 10,038 nulliparous participants enrolled in the original NuMoM2b study, 2971 were excluded due to missing FFQ data, pregnancy loss before 20 weeks of gestation or missing data on key pregnancy outcomes. This left a total of 7067 participants to form the analytic sample in this secondary analysis. Sample sizes for each outcome-specific model reflect complete-case analysis and differ because of outcome-specific missing data: small-for gestational age (SGA) (n = 7067; missing: 0); large for gestational age (n = 6887; missing: 180 (2.5%)); hypertensive disorders of pregnancy (HDP) (n = 7054; missing: 13 (0.2%)); gestational diabetes mellitus (GDM) (n = 7064; missing: 3 (<0.1%)).

Descriptive statistics were used to summarize frequencies and means with their corresponding percentages and standard deviations (SD). In bivariate analysis, Chi square tests were used to compare demographic characteristics, maternal outcomes between quartiles of protein intake. Unadjusted logistic regression models tested the association between protein intake and outcome of interest. Adjusted logistic regression models tested the independent association between protein intake and each of the specified pregnancy outcomes while controlling for potential confounders, which were selected a priori based on clinical relevance or statistical significance [36]. Unadjusted and adjusted odds ratios (aORs) with their corresponding 95% confidence intervals (CIs) were reported. Protein intake was re-analyzed as a continuous exposure using restricted cubic splines (4 knots) within the adjusted logistic regression models and tested for nonlinearity via a joint Wald test of the nonlinear spline terms. In the sensitivity analysis, protein intake was analyzed as a continuous exposure variable using restricted cubic splines with 4 knots placed at the 5th, 35th, 65th, and 95th percentiles of the observed protein intake distribution corresponding with 0.35, 0.68, 0.98, and 1.85 g/kg/day, respectively within adjusted logistic regression models. Missing data were handled using complete-case analysis and no imputation was performed given the low proportion of missing data across all models (Figure 1). All analysis was conducted in STATA/MP v19.5 [37].

## 3. Results

A total of 7067 participants were included in the analysis. Table 1 describes maternal characteristics. The cohort was primarily non-Hispanic White, between 18 and 34 years old, and employed, and held bachelor’s or higher education degrees, with commercial insurance, and with higher income than the federal poverty levels of the time. Women who identified as White fell largely within quartile 2 of a daily protein intake of 0.6–<0.8 g/kg of bodyweight per day. Hispanic and Latina women were more likely to consume a higher daily protein intake of >1.1 g/kg (Table 1). A similar trend was observed among Asian women. However, a lower protein intake of <0.6 g/kg was more common among Black and multiracial women (*p* = 0.001; Table 1). Additionally, women with less than or equal to a high school education were found to have lower protein intake compared to women with higher education (*p* < 0.001, Table 1). Lower protein intake was more prominent among women with a household income below the federal poverty level (*p* = 0.003), unemployment (*p* = 0.004) and government insurance (*p* = 0.036; Table 1).

Table 2 describes the associations between protein intake and pregnancy outcomes. The rates of HDP were higher among women consuming protein in the two lowest quartiles compared to the highest quartile (unadjusted Q1 *p* = 0.02; Q2 *p* = 0.03). After adjusting for potentially confounding factors including maternal age, BMI, insurance, education, poverty level, chronic hypertension, and daily kcal intake, lower protein intake was not significantly associated with higher odds of HDP (adjusted OR 0.96, 95% CI 0.80–1.17 for quartile 1, and adjusted OR 1.12, 95% CI 0.94–1.33 for quartile 2; quartile 4: referent). Of note, significance disappeared after incorporating the daily kcal intake into the regression model (adjusted Q1 *p* = 0.730; Q2 *p* = 0.202). The third quartile for protein intake demonstrated decreased odds of SGA <10th percentile compared to quartile 4 (adjusted OR 0.78, 95% CI 0.61–0.99 for quartile 3; quartile 4: referent). There was no association between GDM or LGA and maternal protein intake.

To test whether the association between protein intake and each pregnancy outcome was linear, protein intake was modeled using restricted cubic splines with four knots in adjusted logistic regression models. The joint significance of the nonlinear spline terms was then tested to determine evidence of a nonlinear association. For SGA < 10th percentile (Table 3, χ^2^(2) = 0.62, *p* = 0.73) and LGA (Table 3, χ^2^(2) = 2.70, *p* = 0.259), the nonlinear spline terms did not significantly improve model fit, indicating no evidence of a nonlinearity association. However, for HDP (Table 3, χ^2^(2) = 10.87, *p* = 0.004) and GDM (Table 3, χ^2^(2) = 7.95, *p* = 0.019), the spline terms were jointly significant, indicating a nonlinear association. Supplemental Figure S1A shows the predicted probability of HDP declining, plateauing, and then declining further as protein intake increased. Predicted probability of GDM increased slightly, decreased, and then rose sharply above 2.0 g/kg of bodyweight per day. A sensitivity analysis was performed using absolute total daily protein intake (grams) in adjusted logistic regression models, excluding BMI category. All adjusted associations were not statistically significant without BMI, suggesting the prior non-linear relationships between protein intake and HDP and GDM may be driven by BMI (Supplemental Table S2).

## 4. Discussion

This secondary analysis examined protein intake and its association with adverse pregnancy outcomes in a cohort of nulliparous women. Although lower protein intake showed an association with an increased risk of HDP, this association was no longer significant after adjustment for total daily dietary intake. The analysis also demonstrated that women who consumed 0.8–≤1.0 g of protein per kilogram of bodyweight (per day) had significantly lower odds of having an SGA infant. However, a consistent dose response across quartiles was not observed. Women with protein intakes ≥ 1.1 g/kg of bodyweight per day were not associated with decreased odds of SGA. Factors we did not assess in this analysis, like protein source and quality, may have influenced these results. Also, higher odds of SGA were not observed in women in the two lowest protein intake quartiles. Thus, restricted cubic analysis was used to examine non-linear associations between protein intake and adverse pregnancy outcomes but found no evidence of this for SGA < 10th percentile or LGA. These data support the original linearity assumption for these outcomes. Overall, lower protein intake was common in women who were Black and women with lower education, unemployment, lower income, and government insurance.

Protein intake is essential for fetal growth and development [16]. The association between protein intake and birthweight has been previously demonstrated in animal models, though the results are conflicting [38,39]. Significantly lower birthweights and reduced sizes were found in pig, rat, and mouse offspring whose mothers were administered low protein diets [38,39,40,41]. In two separate studies, impaired renal development and increased blood pressures were observed in rat offspring exposed to maternal low protein intakes during pregnancy [20,21]. Furthermore, maternal low protein diets were associated with a 20% decrease in rat offspring glomerular numbers, suggesting altered developmental programming of the embryonic kidneys [22]. In hepatic genes, increases in DNA methylation were observed in rat offspring exposed to maternal low protein intake during pregnancy, indicating maternal protein intake may influence offspring epigenetic programming [24,25]. However, folic acid supplementation showed no evidence of hypermethylation, highlighting a protective role for folic acid against epigenetic modifications [24,25]. Conversely, higher protein diets in pregnancy were also associated with lower birthweights in some animal models, while others showed no effect [19,42,43,44]. In another study, high protein intake during pregnancy and lactation in rat models showed elevated blood pressures and glomerulosclerosis in male offspring, whereas female offspring displayed increased food efficiency, bodyweight, and fat pads [19]. These data highlight a relationship between maternal protein intake and offspring health which may be a nonlinear and sex-dependent effect that may pose long-term health consequences for offspring. However, because animal models have unique metabolic and physiological characteristics, human studies are essential to understand and improve maternal–fetal health outcomes in humans [45,46].

Our secondary analysis found an association between protein intake and reduced risk of SGA. This data aligns with previous research. In one study, pregnant Chinese women who consumed higher animal and dairy protein intakes had decreased rates of low birthweights, SGA, and fetal growth restriction [17]. Similarly, in a Danish prospective cohort study, higher protein intake in pregnancy resulted in decreased risk of SGA as well as postpartum weight retention but was associated with increased risk of LGA and offspring overweight/obesity risks later in life [26]. Additionally, a study conducted in Tanzania found that women who consumed high animal protein intake had lower risks of adverse pregnancy outcomes, including SGA [47]. Protein intake may influence fetal growth through the effect of amino acids on key metabolic pathways, including nitric oxide biosynthesis and mammalian target of rapamycin (mTOR) signaling pathway [48,49,50]. Amino acids regulate fetal growth through diverse mechanisms. For example, arginine, which is abundant in meat, soy, nuts, seeds, dairy products, and seafood, serves as a precursor for polyamine and nitric oxide synthesis [50,51]. The conversion of L-arginine to nitric oxide increases nitric oxide bioavailability and promotes vasodilation [51]. This mechanism supports placental angiogenesis and enhances maternal–fetal nutrient exchange, ultimately optimizing fetal growth [49].

While the primary focus of this study is total protein intake, the source of protein may also influence pregnancy outcomes. Prior studies suggest that dietary patterns characterized by higher consumption of red and processed meats are associated with increased risk of SGA infants, whereas diets rich in fish, eggs, and plant-based foods may be protective [52,53]. Limited evidence also indicates that strict vegetarian diets may be associated with higher odds of SGA, though without increased related morbidity [54]. However, data on protein sources in pregnancy remain limited and heterogeneous, highlighting the need for further research to better understand how protein quality and dietary patterns impact pregnancy outcomes.

Our study did not find an association between protein intake and incidence of GDM or LGA, which may be attributed to the low incidence of GDM in the nuMoM2b cohort. One study found that higher intake of plant-based protein decreased the risk of GDM, though the results were not significant [55]. Yet, consuming more animal protein before and during pregnancy was significantly associated with developing GDM [55]. In a separate study, maternal macronutrient intake was assessed in a cohort of pregnant Greek women diagnosed with GDM. Women with a healthy BMI range diagnosed with GDM consumed higher vegetable protein intakes early in pregnancy and demonstrated a significantly higher risk of LGA [56]. In a prospective mother and child cohort study, protein intake > 15% of total dietary intake reduced the risk of SGA but increased the risk of LGA [26]. Conversely, low protein intake, particularly early in pregnancy, has been associated with fetal growth restriction and increased risk of SGA [16,57,58]. These data highlight the intricate balance of protein intake and its varying association with perinatal outcomes related to metabolic health and fetal growth. Though our secondary analysis did not find an association between protein intake and the occurrence of GDM or LGA, perhaps categorizing the protein by source (animal or plant-based) may have identified difference in these outcomes.

Data from the U.S. National Health and Nutrition Examination Survey (NHANES) showed that about 12% of women in the second and third trimesters have inadequate protein intake [59]. Our finding of lower protein intake being more common among socioeconomically vulnerable populations aligns with current knowledge [12,13,60,61,62,63]. One study found lower alternative healthy eating index for pregnancy (AHEI-P) among non-Hispanic Black women and women with low-to-middle incomes [64]. While our results showed Hispanic women consumed higher amounts of protein, when accounting for the intersecting effects of income, a separate study assessing prenatal diet among low-income Hispanic women identified low healthy index scores [65]. While over 60% of women in that study met their protein intake needs, the intake of vegetables, whole grains, fatty acids, and dairy was low, with overconsumption of saturated fats, sodium, and refined sugars [65].

In this secondary analysis, low protein intake was more common among women who were Black and women with high school education or less, low income, unemployment, and reliance on government insurance. These disparities likely reflect broader structural and individual barriers to adequate nutrition during pregnancy, including food insecurity, limited access to healthy foods, cultural influences, and gaps in nutrition education and counseling [12,13,63,64,65,66,67,68,69,70,71,72]. Notably, most associations between protein intake and pregnancy outcomes in our study were not statistically significant, suggesting that factors beyond total protein intake—such as overall diet quality and social determinants of health—may play a more substantial role. These findings underscore the need for clinical and policy-level interventions to improve access to nutritious foods and provide culturally tailored dietary guidance during pregnancy [73,74,75].

The 2025–2030 dietary guidelines for Americans by the U.S. Department of Agriculture (USDA) and U.S. department of Health and Human Services (HHS) warrants close evaluation for their potential public health impacts. Previous guidelines emphasized age-based specific protein targets, and recommended lean or low-fat meat and dairy sources [76,77]. In contrast, the current guidelines broadly recommend higher protein intake (1.2–1.6 g/kg of bodyweight per day), prioritize protein consumption at every meal, and promote greater intake of animal-based protein and full fat dairy products [77,78]. However, data remains limited in showing positive long term health outcomes from higher protein intakes, particularly among pregnant populations [77,79]. While eating iron-rich meats may help prevent anemia during pregnancy, excessive protein intake, particularly red meats, may increase the risks of GDM, HDP, and fetal growth restriction [16,80,81,82,83,84]. In this secondary analysis, higher protein intake was not associated with improved maternal health outcomes. While protein was associated with HDP in unadjusted analysis, after adjusting for overall amount of caloric intake, the association disappeared. These findings highlight the importance of evaluating protein intake within the context of overall maternal nutrition, rather than assuming higher intake provides additional health benefits. Further research is needed to define optimal protein targets to provide informed evidence-based dietary recommendations for pregnant women.

A few limitations of this study should be noted. This was a secondary analysis of the NuMoM2b study; thus, our sample size was limited within the constraints of the original data set. The study population from the original nuMoM2b data set were predominately non-Hispanic White (59.7%) and had overall a higher socioeconomic status. Another limitation was the low rate of GDM in this cohort, limiting our sample size for analyzing the relationship between protein intake and GDM. Also, the FFQ was only administered once during the NuMoM2b study period, in the first trimester. Thus, food frequency data for the second and third trimesters is non-existent. A combination of factors can shape diets in pregnancy like food aversions, cravings, health outcomes, and food accessibility. Assessing how diet and nutrition shifted throughout pregnancy could have provided a more nuanced perspective of perinatal dietary patterns and may have had an influence on the outcomes of interest. We also did not look into the difference between animal or plant-based protein intake; rather, we examined total protein. Protein sources may impact maternal metabolic health uniquely due to the exposure of the type of amino acid profiles, vitamins and fat content. Comparing the intake of animal and plant-based protein sources and their link to adverse health outcomes could identify healthier diets to consider for improved short- and long-term health outcomes. Furthermore, diet patterns, such as frequency and quantity of protein sources, were not included in this secondary analysis as our interest was in assessing daily total protein intake in pregnancy. Food frequency and quantity of protein sources can shape overall diet and may have influenced health outcomes of interest. Alternatively, the effects of low-protein intake may have been compensated for by intake of supplements or an alternative nutrient/food source, which we did not assess in this analysis. Another limitation is that our BMI computations were not age-specific for adolescents hence we may have underestimated or over-estimated BMI.

Lastly, limitations regarding measurement errors should be noted. The FFQ is a self-reported survey and relies heavily on memory for recall, which may result in participants incorrectly reporting food intakes. Also, an over-adjustment or collinearity may have occurred in our results due to our method in expressing protein intake per kilogram of bodyweight and adjusting for BMI in our statistical model. When protein intake is scaled to bodyweight, heavier participants artificially appear to have less protein intake per unit of body mass. When BMI is used to adjust the model, it may erase, or reverse, health outcomes related to protein intake. We included total daily energy intake (kcal) to our adjusted model to account for possible measurement errors. Also, no multiple comparisons were conducted; therefore, future studies are needed to confirm these exploratory findings. Nevertheless, we were able to analyze FFQs in a large demographically diverse pregnancy cohort, providing an insight into the nutritional diets of pregnant women in the United States.

## 5. Conclusions

In conclusion, we found that protein intake varied significantly across maternal socioeconomic characteristics, and moderate to high protein intake during the first trimester was associated with lower odds of SGA in this study population. However, this finding should be considered exploratory given the absence of a dose–response pattern and the null sensitivity analyses. Additional studies are needed to confirm this association and to further clarify the relationship between maternal protein intake and pregnancy outcomes.

## Figures and Tables

**Table 1:** Maternal demographic characteristics by quartiles based on protein intake.

Characteristic	Quartile (by Protein Intake, g/kg)				Total ^1^	*p*-Value
	<0.6 (Q1)	0.6–<0.8 (Q2)	0.8–<1.1 (Q3)	≥1.1 (Q4)	N (%)	
**Population/Quartile**						
**Age**						0.29
13–17	43 (2.4)	32 (1.8)	29 (1.6)	38 (2.2)	142 (2.0)	
18–34	1574 (89.1)	1555 (88.1)	1558 (88.2)	1555 (88.1)	6242 (88.4)	
35–39	127 (7.2)	150 (8.5)	159 (9.0)	141 (7.9)	577 (8.2)	
≥40	23 (1.3)	29 (1.6)	20 (1.1)	32 (1.8)	104 (1.5)	
Body Mass Index (BMI) V1 *						0.465
Underweight	47 (2.7)	36 (2.1)	29 (1.7)	43 (2.5)	155 (2.2)	
Normal weight	920 (52.6)	900 (51.6)	885 (50.9)	887 (51.1)	3592 (51.5)	
Overweight	407 (23.3)	436 (25.0)	450 (25.9)	414 (23.9)	1707 (24.5)	
Obese (Class I)	202 (11.6)	198 (11.4)	198 (11.4)	227 (13.1)	825 (11.8)	
Obese (Class II+)	173 (9.9)	174 (9.9)	176 (10.1)	164 (9.5)	687 (9.9)	
**Ethnicity**						
Hispanic/Latina	289 (16.4)	286 (16.2)	278 (15.7)	346 (19.6)	1199 (16.9)	**0.008**
**Race**						**0.001**
White	1200 (67.9)	1265 (71.6)	1237 (70.0)	1167 (66.1)	4869 (68.9)	
American Indian/ Alaska Native	5 (0.3)	1 (0.1)	4 (0.2)	2 (0.1)	12 (0.17)	
Asian	55 (3.1)	70 (3.9)	85 (4.8)	104 (5.8)	314 (4.44)	
Native Hawaiian/ Other Pacific Islander	6 (0.3)	5 (0.2)	9 (0.5)	8 (0.4)	28 (0.40)	
Black/African American	232 (13.1)	174 (9.8)	193 (10.9)	200 (11.3)	799 (11.3)	
More than one race	112 (6.3)	110 (6.2)	92 (5.2)	102 (5.7)	416 (5.8)	
Unknown/Not reported	157 (8.8)	142 (8.0)	147 (8.3)	183 (10.3)	629 (8.9)	
**Education**						**<0.001**
Bachelor’s or higher	901 (50.9)	978 (55.3)	1013 (57.3)	908 (51.4)	3800 (53.7)	
Some college/Assoc/Tech	506 (28.6)	495 (28.0)	478 (27.0)	531 (30.0)	2010 (28.4)	
≤High school/No diploma	360 (20.3)	293 (16.3)	275 (15.5)	326 (18.4)	1254 (17.7)	
**Employed**						
Yes	1291 (76.6)	1363 (80.5)	1358 (80.2)	1279 (77.0)	5291 (78.6)	**0.004**
**Income (as a percentage of 2013 poverty level)**						
<100%	210 (14.9)	192 (12.7)	201 (13.3)	262 (18.0)	865 (14.7)	**0.003**
100–200%	192 (13.6)	200 (13.3)	205 (13.5)	194 (13.3)	791 (13.4)	
>200%	1007 (71.4)	1112 (73.9)	1102 (73.0)	999 (68.6)	4220 (71.8)	
**Insurance**						**0.036**
Government	482 (27.4)	420 (23.9)	403 (22.9)	475 (26.9)	1780 (25.3)	
Commercial	947 (53.9)	1010 (57.5)	999 (56.8)	956 (54.3)	3912 (55.6)	
Self-pay	301 (17.1)	304 (17.3)	338 (19.2)	306 (17.3)	1249 (17.1)	
**Chronic Hypertension**						
Yes	41 (2.3)	41 (2.3)	53 (3.0)	41 (2.3)	176 (2.5)	0.471

^1^ Data is reported as n (%) within quartiles. Chi square tests examined the association between maternal demographic characteristics, maternal outcomes, and protein intake quartiles. * Visit 1 (V1). Bold *p*-Value represents statistical significance.

**Table 2:** Unadjusted and adjusted logistic regression analysis examining the association between dietary protein intake and pregnancy complications.

Outcome	Quartile (by Protein Intake, g/kg)	N (%)	OR ^1^ (95% CI)	*p*-Value ^1^	aOR ^2^ (95% CI)	*p*-Value ^2^
**Hypertensive disorders of pregnancy (HDP)** n = 1642	≥1.1 (Q4)	372 (21.1)	Ref		Ref	
	0.8–<1.1 (Q3)	413 (23.4)	1.14 [0.98–1.34]	0.1	1.10 [0.92–1.30]	0.291
	**0.6–<0.8 (Q2)**	**426 (24.1)**	**1.19 [1.02–1.39]**	**0.03**	1.12 [0.94–1.33]	0.202
	**<0.6 (Q1)**	**431 (24.4)**	**1.21 [1.03–1.41]**	**0.02**	0.96 [0.80–1.17]	0.730
Gestational diabetes mellitus (GDM) n = 302	≥1.1 (Q4)	79 (4.5)	Ref		Ref	
	0.8–<1.1 (Q3)	76 (4.5)	0.96 [0.70–1.32]	0.8	0.89 [0.63–1.27]	0.545
	0.6–<0.8 (Q2)	84 (4.7)	1.07 [0.78–1.46]	0.69	0.99 [0.70–1.40]	0.967
	<0.6 (Q1)	63 (3.5)	0.79 [0.56–1.11]	0.17	0.75 [0.51–1.10]	0.141
Large for gestational age (LGA) n = 543	≥1.1 (Q4)	144 (8.4)	Ref		Ref	
	0.8–<1.1 (Q3)	142 (8.3)	0.98 [0.77–1.25]	0.90	1.01 [0.78–1.31]	0.947
	0.6–<0.8 (Q2)	131 (7.6)	0.89 [0.70–1.14]	0.38	0.90 [0.69–1.17]	0.426
	<0.6 (Q1)	126 (7.3)	0.85 [0.66–1.09]	0.21	0.94 [0.71–1.24]	0.657
**Small for gestational age (SGA) < 10th percentile** n = 749	≥1.1 (Q4)	185 (10.8)	Ref		Ref	
	**0.8–<1.1 (Q3)**	**181 (10.5)**	0.97 [0.78–1.21]	0.77	**0.78 [0.61–0.99]**	**0.045**
	0.6–<0.8 (Q2)	177 (10.2)	0.95 [0.76–1.18]	0.62	0.84 [0.65–1.08]	0.173
	<0.6 (Q1)	206 (11.9)	1.11 [0.90–1.38]	0.31	0.94 [0.73–1.21]	0.656

^1^ Odds ratio (OR) (95% CI), unadjusted model tested the association between protein intake and each outcome. ^2^ Adjusted odds ratio (aOR) (95% CI) was adjusted for age, BMI, insurance, education, poverty level, chronic hypertension, and total daily intake (kcal). Bold represents statistical significance.

**Table 3:** Multivariable logistic regression test for nonlinear associations between protein intake (g/kg) and pregnancy outcomes using restricted cubic splines.

Outcome	Nonlinearity Test			
	LR χ^2^ (Model)	χ^2^	*p*-Value	Conclusion
Hypertensive disorders of pregnancy (HDP)	202.12	10.87	0.004	Significant nonlinearity
Gestational diabetes mellitus (GDM)	141	7.95	0.019	Significant nonlinearity
Large for gestational age (LGA)	71.43	2.70	0.259	No evidence of nonlinearity
Small for gestational age (SGA) < 10th percentile	70.51	0.62	0.733	No evidence of nonlinearity

Protein intake was modeled continuously using restricted cubic splines with 4 knots within the multivariable logistic regression. The assumption of a linear dose–response relationship was formally tested using a joint Wald test for the nonlinear spline terms.

## Data Availability

The original nuMoM2b data is available in the Eunice Kennedy Shriver National Institute of Child Health and Human Development (NICHD) Data and Specimen Hub (DASH) at https://dash.nichd.nih.gov/ (accessed on 1 January 2025).

## Supplementary Materials

The following supporting information can be downloaded at https://www.mdpi.com/article/10.3390/nu18152543/s1, Table S1. Adjusted restricted cubic spline associations between protein intake (g/kg) and pregnancy outcomes; Table S2. Sensitivity analysis with absolute total daily protein intake in grams, excluding BMI from the analysis; Figure S1. Predicted probability by protein intake with 95% CI.

## Abbreviations

The following abbreviations are used in this manuscript:

FFQ	Food Frequency Questionnaire
nuMoM2b	Nulliparous Pregnancy Outcomes Study: Monitoring Mother-to-Be
g/kg	Grams per kilogram of bodyweight per day
HDP	Hypertensive disorders of pregnancy
GDM	Gestational diabetes mellitus
SGA	Small for gestational age
LGA	Large for gestational age
BMI	Body mass index
OR	Odds ratio
aOR	Adjusted odds ratio
CI	Confidence interval

## References

[1] Talebi S., Kianifar H.R., Mehdizadeh A. (2024). Nutritional requirements in pregnancy and lactation. Clin. Nutr. ESPEN.

[2] Chandra M., Paray A.A. (2024). Natural Physiological Changes During Pregnancy. Yale J. Biol. Med..

[3] Moya J., Phillips L., Sanford J., Wooton M., Gregg A., Schuda L. (2014). A review of physiological and behavioral changes during pregnancy and lactation: Potential exposure factors and data gaps. J. Expo. Sci. Environ. Epidemiol..

[4] Pritschet L., Taylor C.M., Cossio D., Faskowitz J., Santander T., Handwerker D.A., Grotzinger H., Layher E., Chrastil E.R., Jacobs E.G. (2024). Neuroanatomical changes observed over the course of a human pregnancy. Nat. Neurosci..

[5] Pascual Z.N., Langaker M.D. (2025). Physiology, Pregnancy.

[6] Heymsfield S.B., Shapses S.A. (2024). Guidance on Energy and Macronutrients across the Life Span. N. Engl. J. Med..

[7] Marshall N.E., Abrams B., Barbour L.A., Catalano P., Christian P., Friedman J.E., Hay W.W., Hernandez T.L., Krebs N.F., Oken E. (2022). The importance of nutrition in pregnancy and lactation: Lifelong consequences. Am. J. Obstet. Gynecol..

[8] Morales-Suarez-Varela M., Rocha-Velasco O.A. (2025). Impact of ultra-processed food consumption during pregnancy on maternal and child health outcomes: A comprehensive narrative review of the past five years. Clin. Nutr. ESPEN.

[9] Lassi Z.S., Padhani Z.A., Rabbani A., Rind F., Salam R.A., Bhutta Z.A. (2021). Effects of nutritional interventions during pregnancy on birth, child health and development outcomes: A systematic review of evidence from low- and middle-income countries. Campbell Syst. Rev..

[10] Christian P., Mullany L.C., Hurley K.M., Katz J., Black R.E. (2015). Nutrition and maternal, neonatal, and child health. Semin. Perinatol..

[11] Mousa A., Naqash A., Lim S. (2019). Macronutrient and Micronutrient Intake during Pregnancy: An Overview of Recent Evidence. Nutrients.

[12] Kovell L.C., Sibai D., Wilkie G.L., Shankara S., Moinul S., Kaminsky L., Lemon S.C., McManus D.D. (2022). Identifying barriers, facilitators, and interventions to support healthy eating in pregnant women with or at risk for hypertensive disorders of pregnancy. Cardiovasc. Digit. Health J..

[13] O’HAlloran S.A., Sunder P., Cusworth R., Hutchinson A.M., Alston L., Vasilevski V., Sweet L., Olive E., Sominsky L., Vuillermin P. (2025). Midwives’ perspectives on women’s dietary intake during pregnancy: A systems thinking approach. Midwifery.

[14] Espinosa-Salas S., Gonzalez-Arias M. (2025). Nutrition: Macronutrient Intake, Imbalances, and Interventions.

[15] Calvez J., Azzout-Marniche D., Tome D. (2024). Protein quality, nutrition and health. Front. Nutr..

[16] Herring C.M., Bazer F.W., Johnson G.A., Wu G. (2018). Impacts of maternal dietary protein intake on fetal survival, growth, and development. Exp. Biol. Med..

[17] Yang J., Chang Q., Tian X., Zhang B., Zeng L., Yan H., Dang S., Li Y.-H. (2022). Dietary protein intake during pregnancy and birth weight among Chinese pregnant women with low intake of protein. Nutr. Metab..

[18] van Zundert S., van der Padt S., Willemsen S., Rousian M., Mirzaian M., van Schaik R., Steegers-Theunissen R., van Rossem L. (2022). Periconceptional Maternal Protein Intake from Animal and Plant Sources and the Impact on Early and Late Prenatal Growth and Birthweight: The Rotterdam Periconceptional Cohort. Nutrients.

[19] Thone-Reineke C., Kalk P., Dorn M., Klaus S., Simon K., Pfab T., Godes M., Persson P., Unger T., Hocher B. (2006). High-protein nutrition during pregnancy and lactation programs blood pressure, food efficiency, and body weight of the offspring in a sex-dependent manner. Am. J. Physiol. Regul. Integr. Comp. Physiol..

[20] Langley S.C., Jackson A.A. (1994). Increased systolic blood pressure in adult rats induced by fetal exposure to maternal low protein diets. Clin. Sci..

[21] Woods L.L., Weeks D.A., Rasch R. (2004). Programming of adult blood pressure by maternal protein restriction: Role of nephrogenesis. Kidney Int..

[22] Welham S.J., Riley P.R., Wade A., Hubank M., Woolf A.S. (2005). Maternal diet programs embryonic kidney gene expression. Physiol. Genom..

[23] Geraghty A.A., Lindsay K.L., Alberdi G., McAuliffe F.M., Gibney E.R. (2015). Nutrition During Pregnancy Impacts Offspring’s Epigenetic Status-Evidence from Human and Animal Studies. Nutr. Metab. Insights.

[24] Lillycrop K.A., Phillips E.S., Jackson A.A., Hanson M.A., Burdge G.C. (2005). Dietary protein restriction of pregnant rats induces and folic acid supplementation prevents epigenetic modification of hepatic gene expression in the offspring. J. Nutr..

[25] Gong L., Pan Y.X., Chen H. (2010). Gestational low protein diet in the rat mediates Igf2 gene expression in male offspring via altered hepatic DNA methylation. Epigenetics.

[26] Zhang H., Senior A.M., Saner C., A Koemel N., Simpson S.J., Raubenheimer D., Heitmann B.L. (2025). Maternal protein intake during pregnancy and obesity risk in mothers and offspring: A prospective cohort study. Am. J. Clin. Nutr..

[27] (1998). Dietary Reference Intakes: A Risk Assessment Model for Establishing Upper Intake Levels for Nutrients.

[28] Elango R., Ball R.O. (2016). Protein and Amino Acid Requirements during Pregnancy. Adv. Nutr..

[29] Trumbo P., Schlicker S., Yates A.A., Poos M., Food and Nutrition Board of the Institute of Medicine, The National Academies (2002). Dietary reference intakes for energy, carbohydrate, fiber, fat, fatty acids, cholesterol, protein and amino acids. J. Am. Diet. Assoc..

[30] Stephens T.V., Woo H., Innis S.M., Elango R. (2014). Healthy pregnant women in Canada are consuming more dietary protein at 16- and 36-week gestation than currently recommended by the Dietary Reference Intakes, primarily from dairy food sources. Nutr. Res..

[31] Haas D.M., Parker C.B., Wing D.A., Parry S., Grobman W.A., Mercer B.M., Simhan H.N., Hoffman M.K., Silver R.M., Wadhwa P. (2015). A description of the methods of the Nulliparous Pregnancy Outcomes Study: Monitoring mothers-to-be (nuMoM2b). Am. J. Obstet. Gynecol..

[32] Block G., Woods M., Potosky A., Clifford C. (1990). Validation of a self-administered diet history questionnaire using multiple diet records. J. Clin. Epidemiol..

[33] Hauspurg A., Marsh D.J., McNeil R.B., Merz C.N.B., Greenland P., Straub A.C., Rouse C.E., Grobman W.A., Pemberton V.L., Silver R.M. (2022). Association of N-Terminal Pro-Brain Natriuretic Peptide Concentration in Early Pregnancy With Development of Hypertensive Disorders of Pregnancy and Future Hypertension. JAMA Cardiol..

[34] Facco F.L.M., Parker C.B.D., Reddy U.M., Silver R.M., Koch M.A., Louis J.M., Basner R.C., Chung J.H., Nhan-Chang C.-L., Pien G.W.M. (2017). Association Between Sleep-Disordered Breathing and Hypertensive Disorders of Pregnancy and Gestational Diabetes Mellitus. Obstet. Gynecol..

[35] Talge N.M., Mudd L.M., Sikorskii A., Basso O. (2014). United States birth weight reference corrected for implausible gestational age estimates. Pediatrics.

[36] American College of Obstetricians and Gynecologists’ Committee on Practice Bulletins—Obstetrics (2019). ACOG Practice Bulletin No. 203: Chronic Hypertension in Pregnancy. Obstet. Gynecol..

[37] StataCorp (2025). Stata Statistical Software: Release 19.

[38] Altmann S., Murani E., Metges C.C., Schwerin M., Wimmers K., Ponsuksili S. (2012). Effect of gestational protein deficiency and excess on hepatic expression of genes related to cell cycle and proliferation in offspring from late gestation to finishing phase in pig. Mol. Biol. Rep..

[39] Oster M., Murani E., Metges C.C., Ponsuksili S., Wimmers K. (2012). A low protein diet during pregnancy provokes a lasting shift of hepatic expression of genes related to cell cycle throughout ontogenesis in a porcine model. BMC Genom..

[40] Folguieri M.S., Calsa B., Boer P.A., Gontijo J.A.R. (2025). The earliest impact of restricted protein intake during pregnancy on heart development in male mouse offspring. Front. Cell Dev. Biol..

[41] Mesquita F.F., Gontijo J.A., Boer P.A. (2010). Expression of renin-angiotensin system signalling compounds in maternal protein-restricted rats: Effect on renal sodium excretion and blood pressure. Nephrol. Dial. Transplant..

[42] Oster M., Murani E., Metges C.C., Ponsuksili S., Wimmers K. (2011). A high protein diet during pregnancy affects hepatic gene expression of energy sensing pathways along ontogenesis in a porcine model. PLoS ONE.

[43] Carlin G., Chaumontet C., Blachier F., Barbillon P., Darcel N., Blais A., Delteil C., Guillin F.M., Blat S., Van der Beek E.M. (2019). Maternal High-Protein Diet during Pregnancy Modifies Rat Offspring Body Weight and Insulin Signalling but Not Macronutrient Preference in Adulthood. Nutrients.

[44] de Maredsous C.D., Oozeer R., Barbillon P., Mary-Huard T., Delteil C., Blachier F., Tomé D., van der Beek E.M., Davila A.-M. (2016). High-Protein Exposure during Gestation or Lactation or after Weaning Has a Period-Specific Signature on Rat Pup Weight, Adiposity, Food Intake, and Glucose Homeostasis up to 6 Weeks of Age. J. Nutr..

[45] Michonska I., Luszczki E., Zielinska M., Oleksy L., Stolarczyk A., Deren K. (2022). Nutritional Programming: History, Hypotheses, and the Role of Prenatal Factors in the Prevention of Metabolic Diseases-A Narrative Review. Nutrients.

[46] Hsu C.N., Tain Y.L. (2019). The Good, the Bad, and the Ugly of Pregnancy Nutrients and Developmental Programming of Adult Disease. Nutrients.

[47] Kamenju P., Madzorera I., Hertzmark E., Urassa W., Fawzi W.W. (2022). Higher Dietary Intake of Animal Protein Foods in Pregnancy Is Associated with Lower Risk of Adverse Birth Outcomes. J. Nutr..

[48] Hussain T., Tan B., Murtaza G., Metwally E., Yang H., Kalhoro M.S., Kalhoro D.H., Chughtai M.I., Yin Y. (2020). Role of Dietary Amino Acids and Nutrient Sensing System in Pregnancy Associated Disorders. Front. Pharmacol..

[49] Wu G., Bazer F.W., Satterfield M.C., Li X., Wang X., Johnson G.A., Burghardt R.C., Dai Z., Wang J., Wu Z. (2013). Impacts of arginine nutrition on embryonic and fetal development in mammals. Amino Acids.

[50] Tain Y.L., Hsu C.N. (2024). Amino Acids during Pregnancy and Offspring Cardiovascular-Kidney-Metabolic Health. Nutrients.

[51] Vasdev S., Stuckless J. (2010). Antihypertensive effects of dietary protein and its mechanism. Int. J. Angiol..

[52] Ricci E., Chiaffarino F., Cipriani S., Malvezzi M., Parazzini F. (2010). Diet in pregnancy and risk of small for gestational age birth: Results from a retrospective case-control study in Italy. Matern. Child Nutr..

[53] Knudsen V.K., Orozova-Bekkevold I.M., Mikkelsen T.B., Wolff S., Olsen S.F. (2008). Major dietary patterns in pregnancy and fetal growth. Eur. J. Clin. Nutr..

[54] Yisahak S.F., Hinkle S.N., Mumford S.L., Li M., Andriessen V.C., Grantz K.L., Zhang C., Grewal J. (2021). Vegetarian diets during pregnancy, and maternal and neonatal outcomes. Int. J. Epidemiol..

[55] Tabaeifard R., Moradi M., Arzhang P., Azadbakht L. (2024). Association between protein intake and risk of gestational diabetes mellitus: A systematic review and dose-response meta-analysis of cohort studies. Clin. Nutr..

[56] Siargkas A., Tranidou A., Magriplis E., Tsakiridis I., Apostolopoulou A., Xenidis T., Pazaras N., Chourdakis M., Dagklis T. (2025). Impact of Maternal Macronutrient Intake on Large for Gestational Age Neonates’ Risk Among Women with Gestational Diabetes Mellitus: Results from the Greek BORN2020 Cohort. Nutrients.

[57] Zhao F., Du W.Q., Shen J.X., Guo L.L., Wang Y., Wang K.K., Zhang P., Feng Y.L., Yang H.L., Wang S.P. (2019). Association between maternal dietary intake and the incidence of babies with small for gestational age. Zhonghua Liu Xing Bing Xue Za Zhi.

[58] Ji Y., Wu Z., Dai Z., Wang X., Li J., Wang B., Wu G. (2017). Fetal and neonatal programming of postnatal growth and feed efficiency in swine. J. Anim. Sci. Biotechnol..

[59] Murphy M.M., Higgins K.A., Bi X., Barraj L.M. (2021). Adequacy and Sources of Protein Intake among Pregnant Women in the United States, NHANES 2003–2012. Nutrients.

[60] Murphy R., Marshall K., Zagorin S., Devarshi P.P., Hazels Mitmesser S. (2022). Socioeconomic Inequalities Impact the Ability of Pregnant Women and Women of Childbearing Age to Consume Nutrients Needed for Neurodevelopment: An Analysis of NHANES 2007–2018. Nutrients.

[61] Marian M., Pérez R.L., McClain A.C., Hurst S., Reed E., Barker K.M., Lundgren R. (2025). Nutritional knowledge and practices of low-income women during pregnancy: A qualitative study in two Oaxacan cities. J. Health Popul. Nutr..

[62] Serbesa M.L., Iffa M.T., Geleto M. (2019). Factors associated with malnutrition among pregnant women and lactating mothers in Miesso Health Center, Ethiopia. Eur. J. Midwifery.

[63] Girardi G., Longo M., Bremer A.A. (2023). Social determinants of health in pregnant individuals from underrepresented, understudied, and underreported populations in the United States. Int. J. Equity Health.

[64] Parker H.W., Tovar A., McCurdy K., Vadiveloo M. (2020). Socio-economic and racial prenatal diet quality disparities in a national US sample. Public Health Nutr..

[65] Thomas Berube L., Messito M.J., Woolf K., Deierlein A., Gross R. (2019). Correlates of Prenatal Diet Quality in Low-Income Hispanic Women. J. Acad. Nutr. Diet..

[66] Kavle J.A., Landry M. (2018). Addressing barriers to maternal nutrition in low- and middle-income countries: A review of the evidence and programme implications. Matern. Child Nutr..

[67] de Diego-Cordero R., Rivilla-Garcia E., Diaz-Jimenez D., Lucchetti G., Badanta B. (2021). The role of cultural beliefs on eating patterns and food practices among pregnant women: A systematic review. Nutr. Rev..

[68] Martin S.L., McCann J.K., Gascoigne E., Allotey D., Fundira D., Dickin K.L. (2021). Engaging family members in maternal, infant and young child nutrition activities in low- and middle-income countries: A systematic scoping review. Matern. Child Nutr..

[69] Jhaveri N.R., Poveda N.E., Kachwaha S., Comeau D.L., Nguyen P.H., Young M.F. (2023). Opportunities and barriers for maternal nutrition behavior change: An in-depth qualitative analysis of pregnant women and their families in Uttar Pradesh, India. Front. Nutr..

[70] Vander Wyst K.B., Quintana G., Balducci J., Whisner C.M. (2019). Comparison and Characterization of Prenatal Nutrition Counseling among Large-for-Gestational Age Deliveries by Pre-Pregnancy BMI. Nutrients.

[71] Lucas C., Charlton K.E., Yeatman H. (2014). Nutrition advice during pregnancy: Do women receive it and can health professionals provide it?. Matern. Child Health J..

[72] Quayyum F., Dombrowski S.U. (2021). Barriers to nutritional pregnancy preparation and support needs in women and men: Qualitative study based on the Theoretical Domains Framework. Womens Health.

[73] Dewidar O., John J., Baqar A., Madani M.T., Saad A., Riddle A., Ota E., Kung’U J.K., Arabi M., Raut M.K. (2023). Effectiveness of nutrition counseling for pregnant women in low- and middle-income countries to improve maternal and infant behavioral, nutritional, and health outcomes: A systematic review. Campbell Syst. Rev..

[74] Rainford M., Barbour L.A., Birch D., Catalano P., Daniels E., Gremont C., Marshall N.E., Wharton K., Thornburg K. (2024). Barriers to implementing good nutrition in pregnancy and early childhood: Creating equitable national solutions. Ann. N. Y. Acad. Sci..

[75] Caut C., Leach M., Steel A. (2020). Dietary guideline adherence during preconception and pregnancy: A systematic review. Matern. Child Nutr..

[76] United States Department of Agriculture and United States Department of Health and Human Resources (2020). Dietary Guidelines for Americans, 2020–2025.

[77] Papandreou D., Alsuwaidi A., Taha Z., Giaginis C., Vasios G.K., Andreou E.P. (2026). A Critical Narrative Review Appraisal of the 2025–2030 Dietary Guidelines: Scientific Strengths, Conceptual Gaps, and Overlooked Dimensions of Sustainability and Health Equity. Nutrients.

[78] United States Department of Agriculture and United States Department of Health and Human Resources (2025). Dietary Guidelines for Americans, 2025–2030.

[79] Vogtschmidt Y.D., Raben A., Faber I., de Wilde C., Lovegrove J.A., Givens D.I., Pfeiffer A.F., Soedamah-Muthu S.S. (2021). Is protein the forgotten ingredient: Effects of higher compared to lower protein diets on cardiometabolic risk factors. A systematic review and meta-analysis of randomized controlled trials. Atherosclerosis.

[80] Quan W., Zeng M., Jiao Y., Li Y., Xue C., Liu G., Wang Z., Qin F., He Z., Chen J. (2021). Western Dietary Patterns, Foods, and Risk of Gestational Diabetes Mellitus: A Systematic Review and Meta-Analysis of Prospective Cohort Studies. Adv. Nutr..

[81] Kibret K.T., Chojenta C., Gresham E., Tegegne T.K., Loxton D. (2019). Maternal dietary patterns and risk of adverse pregnancy (hypertensive disorders of pregnancy and gestational diabetes mellitus) and birth (preterm birth and low birth weight) outcomes: A systematic review and meta-analysis. Public Health Nutr..

[82] Zhang C., Schulze M.B., Solomon C.G., Hu F.B. (2006). A prospective study of dietary patterns, meat intake and the risk of gestational diabetes mellitus. Diabetologia.

[83] (2021). Anemia in Pregnancy: ACOG Practice Bulletin, Number 233. Obstet. Gynecol..

[84] Mayo Clinic Iron Deficiency Anemia During Pregnancy: Prevention Tips 2025. https://www.mayoclinic.org/healthy-lifestyle/pregnancy-week-by-week/in-depth/anemia-during-pregnancy/art-20114455.

