# Supplementary material for: Association Between Maternal Protein Intake and the Risk of Adverse Pregnancy Outcomes
Source: Nutrients. 2026 Aug 4;18(15):2543. doi: 10.3390/nu18152543 (PMC13468393; doi:10.3390/nu18152543)
Supplement: Supplementary file 1 [file nutrients-18-02543-s001.zip › nutrients-4437717-supplementary.pdf]

Supplementary Table S1. Adjusted restricted cubic spline associations between protein intake (g/kg) and pregnancy outcomes

Outcome	Spline Term	βCoefficient	Std. Error	p-value	P-value for Nonlinearity*
Hypertensive disorders of pregnancy (HDP)	prot_rcs1 (Linear)	-1.56	0.43	<0.001	**0.004**
	prot_rcs2 (Nonlinear)	5.26	2.20	0.017	
	prot_rcs3 (Nonlinear)	-11.69	5.33	0.028	
Gestational diabetes mellitus (GDM)	prot_rcs1 (Linear)	2.12	0.92	0.021	**0.019**
	prot_rcs2 (Nonlinear)	-12.37	4.63	0.008	
	prot_rcs3 (Nonlinear)	30.31	11.1	0.006	
Large for gestational age (LGA)	prot_rcs1 (Linear)	-1.72	0.66	0.009	0.259
	prot_rcs2 (Nonlinear)	5.42	3.47	0.118	
	prot_rcs3 (Nonlinear)	-12.92	8.61	0.133	
Small for gestational age (SGA) <10th percentile	prot_rcs1 (Linear)	0.14	0.63	0.823	0.733
	prot_rcs2 (Nonlinear)	2.38	3.1	0.443	
	prot_rcs3 (Nonlinear)	-5.59	7.44	0.453	

* Bold indicates significance.

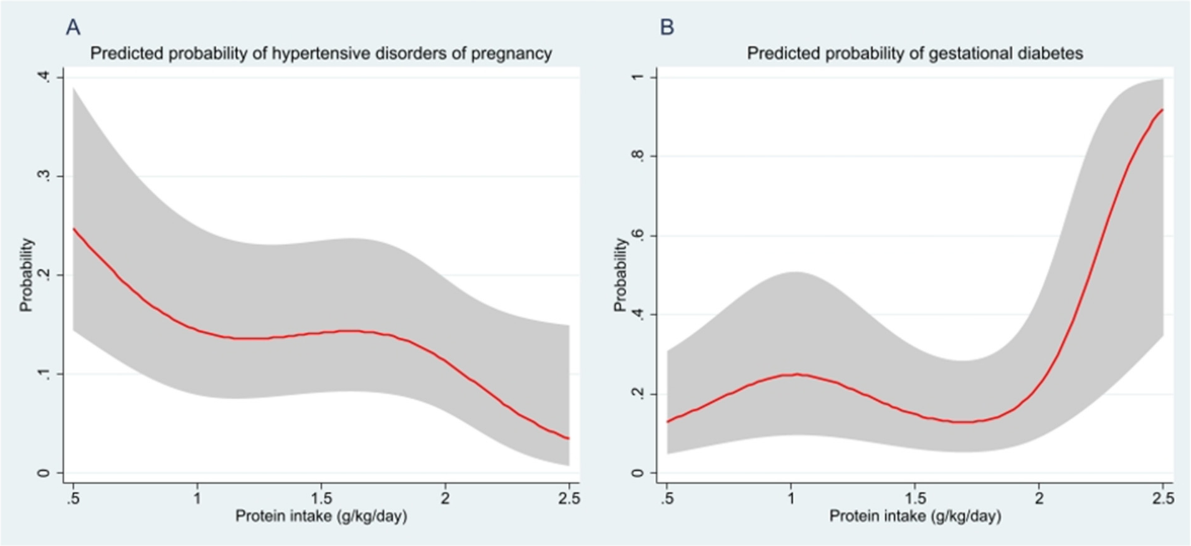

Supplemental Figure S1. Predicted probability by protein intake with 95% CI. In order to test whether the association between maternal protein intake and each pregnancy outcome was linear, we modeled protein intake using restricted cubic splines (4 knots) within adjusted logistic regression models and tested the joint significance of the nonlinear spline terms. For SGA <10th percentile thresholds ($\chi^2(2)=0.62$, p=0.73), and for LGA ($\chi^2(2)=2.70$, p=0.259), the nonlinear terms did not significantly improve model fit, suggesting no evidence of nonlinearity between protein intake and the respective outcomes. However, for HDP ($\chi^2(2)=10.87$, p=0.004) and GDM ($\chi^2(2)=7.95$, p=0.019), the spline terms were jointly significant, indicating the association between protein intake and these two outcomes (HDP and GDM) is non-linear relationship. Figure S1A shows predicted probability of HDP declining, plateauing then further decline with increase in protein intake. In addition, predicted probability of GDM (Figure S1B) had slight increase then decrease before increasing sharply above 2.0 g/kg/day.

Supplemental Table S2. Sensitivity analysis with absolute total daily protein intake in grams, excluding BMI from the analysis.

Outcome	OR*	95% CI	p-value
Hypertensive disorders of pregnancy (HDP)	1	0.99-1.03	0.383
Gestational diabetes mellitus (GDM)	1.01	0.99-1.01	0.179
Large for gestational age (LGA)	1	0.99-1.01	0.256
Small for gestational age (SGA) <10th percentile	1	0.99-1.01	0.882

*Odds ratio (OR); 95% CI = 95% confidence interval. Multivariable logistic regression models adjusted for maternal age category, education, poverty category, chronic hypertension, and total daily energy intake (kcal). BMI category was excluded to avoid collinearity with the prior weight-based exposure used.
